# Health system strengthening recommendations for lung cancer care: lessons from the COVID-19 pandemic

**DOI:** 10.1093/oncolo/oyag144

**Published:** 2026-04-16

**Authors:** Elysse Bautista-González, Mercedes Aguerrebere, Anne Peasey, Hynek Pikhart, Cecilia Vindrola-Padros

**Affiliations:** Institute of Epidemiology and Health Care, University College London, London, WC1E 7HB, United Kingdom; Department of Global Health and Social Medicine, Harvard Medical School, Boston, MA 02115, United States; Institute of Epidemiology and Health Care, University College London, London, WC1E 7HB, United Kingdom; Institute of Epidemiology and Health Care, University College London, London, WC1E 7HB, United Kingdom; Department of Targeted Intervention, University College London, Charles Bell House, London, W1W 7TY, United Kingdom

**Keywords:** lung cancer, COVID-19, health services research

## Abstract

We explore how the COVID-19 pandemic amplified barriers across the lung cancer care continuum in Mexico. Drawing from a cross-sectional qualitative arm of a mixed-methods study (*N* = 46), interviews were conducted with lung cancer patients at the Mexican National Cancer Institute between 2020 and 2021. Using thematic analysis, patients reported care delays due to fear of infection, unclear communication, and service disruptions. Thematic patterns revealed that these challenges were compounded by financial hardship, emotional distress, and reluctance to seek care. Key policy and practice implications include the need for flexible healthcare delivery models, improved communication strategies, financial support mechanisms, tailored interventions for care-seeking behaviors, and integrated mental health and community support. These findings underscore the urgency for systemic adaptations to safeguard timely cancer care during future health crises, particularly in middle-income settings like Mexico. Ethical approval was obtained from the Mexican National Cancer Institute.

Lung cancer patients participated in structured interviews on symptom recognition, healthcare-seeking behavior, and the impact of the COVID-19 pandemic. Interviews were analyzed thematically using Braun and Clarke’s framework. The following themes arose:

## Symptom overlap and diagnostic delays in lung cancer

The pandemic had mixed effects on people’s healthcare-seeking behaviors.^1^ In Mexico, on one hand, the resemblance of respiratory signs and symptoms associated with both coronavirus and lung cancer led several patients to seek medical attention with the suspicion of having contracted COVID-19 or due to presumed sequelae from a prior infection. Following a careful differential diagnosis, these individuals were subsequently diagnosed with lung cancer, a condition that might have otherwise gone unnoticed. Conversely, other patients opted to defer seeking medical attention for specific respiratory symptoms, attributing them to potential sequelae of a recent COVID-19 infection. This delay in seeking medical care resulted in a prolonged period before the diagnosis.

## Continuity of cancer care in a disrupted health system and the erosion of healthcare trust

Around the world the COVID-19 pandemic caused significant disruptions in healthcare delivery.^2–4^ In lung cancer, particularly, patients experienced modifications to treatment plans to reduce the risk of COVID-19 exposure. This led to compromising survival outcomes and reducing their mental health.^5^ Although some trust was deposited in the Mexican government during the rollout of vaccination initiated in 2021, mistrust in the healthcare system increased as uncertainty in the pathways to care became evident. At a system level, public providers interrupted or deferred care due to hospital conversion to COVID-19 care centers, while some private ones closed. Then patients had to be referred to another hospital, interrupting the pathway to treatment and ultimately delaying care. As a result, increasing negative effects, either through a declining number of registered patients, increased drop-outs and reduced treatment rates, were reported.^6–8^

## Out-of-pocket costs

Expenses to reduce the risk of becoming infected in hospitals were made (i.e., face shields, gloves, and face masks) for patients and family members. In some cases, patients and family members lost their jobs due to COVID-19 contingency measures, and therefore households were under pressure to maintain both the family’s economy and the out-of-pocket expenditures related to cancer care. As a result, insufficient money for treatment and related expenses increased stress and frustration, considering giving up cancer care.

## Isolation and mental health challenges among lung cancer patients

Feelings of fear, guilt, and distress acted across all previous barriers. Either because of the household tasks, out-of-pocket expenses, isolation, self-neglect, or fear of family COVID-19 contagion, patients experienced an additional burden of distress to cancer. For example, relatives who were younger and healthier were those asked to collect medicines from the hospital so cancer patients could stay safe at home. Public transport was avoided, and people preferred private transportation to reduce chances of “catching the virus.” These kinds of situations generated greater social suffering and mental distress for lung cancer patients as they felt guilty about also becoming an economic burden.

While being considered a high-risk population not only by their family but also by themselves, patients were frequently isolated to prevent infection with COVID-19. Being away from social life caused patient sadness and frustration, especially during the peaks of the epidemiological waves. Some patients mentioned being particularly affected by not being able to be with their grandchildren. In some cases, patients, despite realizing their health was at stake, preferred not to be a burden and avoided asking their family members for help. They decided not to speak or delayed speaking about their symptoms until they became unbearable. Patients were not only worried about becoming infected but also reported being afraid of bringing their relatives to the medical appointments, as they would also feel guilty if any of their family members were to become infected with COVID-19.

## Public health messaging for informed cancer care during crises

Once the lung cancer diagnosis was established, fear became amplified by continuous exposure to pandemic-related healthcare news that underscored the elevated likelihood of severe COVID-19 cases in individuals with underlying health issues, including cancer. Interestingly, many patients actively chose to disengage from pandemic news as a self-care measure, aiming to mitigate feelings of fear, anxiety, and uncertainty surrounding the evolving situation. Conversely, others adopted a proactive approach, staying informed about the pandemic’s status and the progress of vaccination campaigns. For this group, such informationserved as a valuable tool, enabling them to make informed decisions about resuming their cancer treatment, including identifying suitable timelines based on the pandemic’s trajectory.

## Discussion

From the policy perspective, health system preparedness policies to ensure early diagnosis of symptomatic cancer during health crises are necessary. For example, policies in the future should focus on strengthening the healthcare system to ensure continuity of care for chronic diseases during crises.^9^ This involves creating flexible frameworks that allow for rapid adaptation of medical facilities, resources, and personnel based on the evolving needs of the community. Integrated care models that consider the interconnectedness of physical and mental health should be prioritized to provide comprehensive support for patients facing both COVID-19 and other health challenges.

Innovative financial mechanisms must be put in place to ensure that economic constraints do not become barriers for cancer care during the crisis, and addressing the mental health needs of these vulnerable populations should be an integral component of future crises. Healthcare providers should place the highest importance on the integration of remote mental monitoring and support into routine lung cancer care.

It is critical to have clear and accessible public health communications during crises.^9^ Messaging should be tailored to cancer patients and delivered through trusted, reliable channels to empower individuals to make timely healthcare decisions and avoid delays in diagnosis or treatment. This includes targeted efforts to improve symptom recognition, promote early care-seeking, support patients in navigating the healthcare system,^4^ as well as. maintaining transparent communication to preserve trust in health services, especially during periods of disruption and uncertainty.^10^

## Conclusion

Health systems should consider our recommendations to be more prepared in future crisis responses.

## Data Availability

The data underlying this article cannot be shared publicly due to the privacy of individuals that participated in the study.
